# A novel method for neck coordination exercise – a pilot study on persons with chronic non-specific neck pain

**DOI:** 10.1186/1743-0003-5-36

**Published:** 2008-12-23

**Authors:** Ulrik Röijezon, Martin Björklund, Mikael Bergenheim, Mats Djupsjöbacka

**Affiliations:** 1 Centre for Musculoskeletal Research, University of Gävle, Gävle, Sweden; 2 Department of Community Medicine and Rehabilitation, Physiotherapy, University of Umeå, Umeå, Sweden; 3 Alfta Research Foundation, Alfta, Sweden; 4 Department of Surgery, Central Hospital Karlstad, Karlstad, Sweden

## Abstract

**Background:**

Chronic neck pain is a common problem and is often associated with changes in sensorimotor functions, such as reduced proprioceptive acuity of the neck, altered coordination of the cervical muscles, and increased postural sway. In line with these findings there are studies supporting the efficacy of exercises targeting different aspects of sensorimotor function, for example training aimed at improving proprioception and muscle coordination. To further develop this type of exercises we have designed a novel device and method for neck coordination training. The aim of the study was to investigate the clinical applicability of the method and to obtain indications of preliminary effects on sensorimotor functions, symptoms and self-rated characteristics in non-specific chronic neck pain

**Methods:**

The study was designed as an uncontrolled clinical trial including fourteen subjects with chronic non-specific neck pain. A new device was designed to allow for an open skills task with adjustable difficulty. With visual feedback, subjects had to control the movement of a metal ball on a flat surface with a rim strapped on the subjects' head. Eight training sessions were performed over a four week period. Skill acquisition was measured throughout the intervention period. After intervention subjects were interviewed about their experience of the exercise and pain and sensorimotor functions, including the fast and slow components of postural sway and jerkiness-, range-, position sense-, movement time- and velocity of cervical rotation, were measured. At six-month follow up, self-rated pain, health and functioning was collected.

**Results:**

The subjects improved their skill to perform the exercise and were overall positive to the method. No residual negative side-effects due to the exercise were reported. After intervention the fast component of postural sway (p = 0.019) and jerkiness of cervical rotation (p = 0.032) were reduced. The follow up showed decreased disability (one out of three indices) and fear of movement, and increased general health (three out of eight dimensions).

**Conclusion:**

The results support the clinical applicability of the method. The improvements in sensorimotor functions may suggest transfer from the exercise to other, non-task specific motor functions and justifies a future randomized controlled trial.

## Background

Chronic neck pain is a common problem [1]. Since the knowledge on the pathophysiology is scarce, treatment efforts are largely pragmatic, focusing on pain management and restoring functioning and abilities. In order to advance the efficacy of treatment and rehabilitation, new knowledge has to be integrated in clinical practice. One area that has generated much novel results over the last years is the research on sensorimotor functions in chronic neck pain. Thus, a wide range of changes in sensorimotor functions have been identified in these conditions, such as: reduced proprioceptive sensibility of the neck [2,3] and shoulder [4]; increased jerkiness of cervical rotations [3]; impaired postural control in quiet stance [5-8]; and altered activation patterns of cervical muscles [9]. Based on such evidence from clinical research, along with data from experimental studies, several models on the pathogenesis of chronic musculoskeletal pain conditions includes mechanisms involving sensorimotor functions (e.g., [10,11]).

In line with the above, studies on neck pain patients evaluating the efficacy of exercise regimes targeting cervical sensorimotor functions have shown promising results. Thus, specific exercises for eye-head-neck coordination were shown to reduce pain [12-14] as well as to improve kinaesthetic sense and cervical range of movement [12,14] at retests after interventions. To improve the neuromuscular coordination of the deep ventral cervical muscles, a specific cranio-cervical flexion exercise has been developed together with an adherent device. This exercise was shown to reduce headache in patients suffering from cervicogenic headache [15] as well as to reduce pain and improve kinaesthetic sense in chronic neck pain patients [14].

The above mentioned exercises focus on slow movements and closed skills tasks (i.e., the task is highly predictable). Drawing on the literature on motor learning, an opportunity for further development of exercises targeting sensorimotor function of the neck resides in designing an exercise which entails a more open skills task, for example via an unstable dynamic system. Such an exercise is less predictable and demand on-line adjustments of the neuromuscular control system. The exercise should preferably be performed in a functional position and include active problem solving, feedback of results as well as progression of difficulty in order to promote motor learning [16]. To achieve persistence of the exercise effects, as well as transfer to different tasks and task contexts, it is vital to implement components of variation and cognitive effort [17]. These components are partly inherent in open skills tasks. Lastly, to be applicable in a clinical setting, the exercise task needs to be easy to understand for the patient, the equipment convenient to use and the level of task difficulty adjustable to suit the individual patient's skill level. Based on these theoretical and empirical premises we judged that the development of novel methods for neck coordination exercises is important in order to improve the rehabilitation of people suffering from chronic neck pain.

The present paper describes a novel exercise method aiming at improving sensorimotor functioning of the neck, and a pilot study of its clinical applicability and preliminary indications of its efficacy for persons with chronic non-specific neck pain. The specific aims were to study the skill acquisition and to assess the subjects experiences of the exercise. A further aim was to find out preliminary information about changes in sensorimotor functions, pain and self-reported characteristics after a four week exercise period with the novel method.

## Methods

The study was designed as an uncontrolled clinical trial. Baseline measurements and post intervention measurements were collected within a week before and a week after the intervention, respectively. Follow up measurements were collected six months after the last exercise session. The study was approved by the Regional Ethical Review Board in Uppsala and written informed consent was obtained from all subjects before the start of the trial.

### Subjects

Seventeen subjects were recruited from a vocational rehabilitation center (Alfta Rehab Center, Sweden) and by advertising in the surrounding area. The inclusion criteria were non-traumatic neck pain, location confirmed with pain drawings [18], with a duration of at least three months, age of 18–50 years and a disability score of 10 or more on the Neck Disability Index (NDI) (scale 0–100) [19]. Subjects were excluded if they had evidence of surgical operation or injury with fracture or luxation of the spine or shoulder, neurological or rheumatic disease, or if they were unable to rotate the head 25° bilaterally or balance a light flat pillow on the head for five seconds (s). Three subjects were excluded from the analysis; two due to misclassification of the NDI scores at the inclusion and one for whom neck trauma was discovered during the interview after commencing the trial. Thus the results are based on 14 subjects (10 females), with the mean age of 35 (SD 10) years, and a median pain duration of 60 months (IQR, 26.5–118).

### Treatment intervention

**Figure 1:**
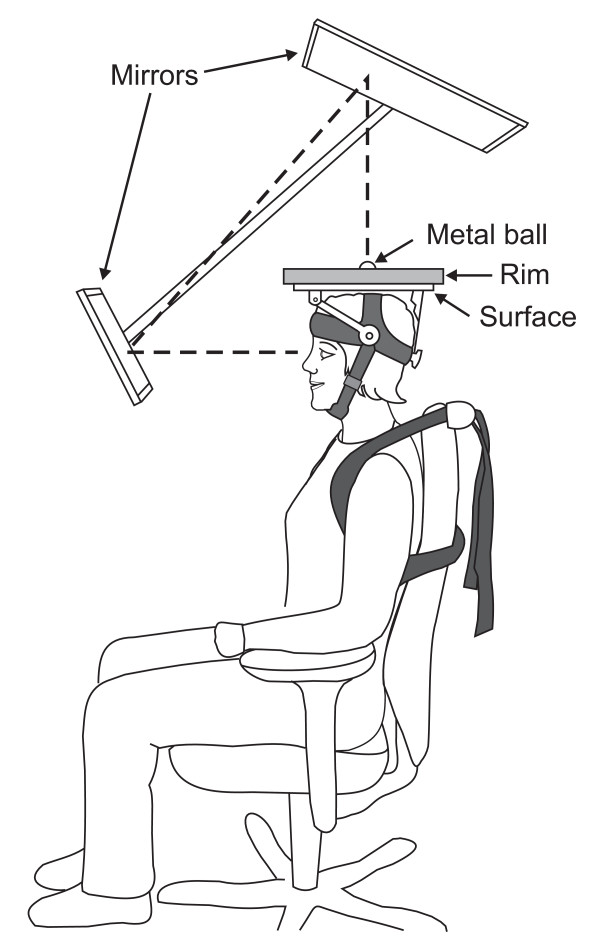
**Schematic of the neck coordination exercise apparatus**. The exercise task was to control the movement of a metal ball on a flat surface mounted on the subjects head.

**Figure 2:**
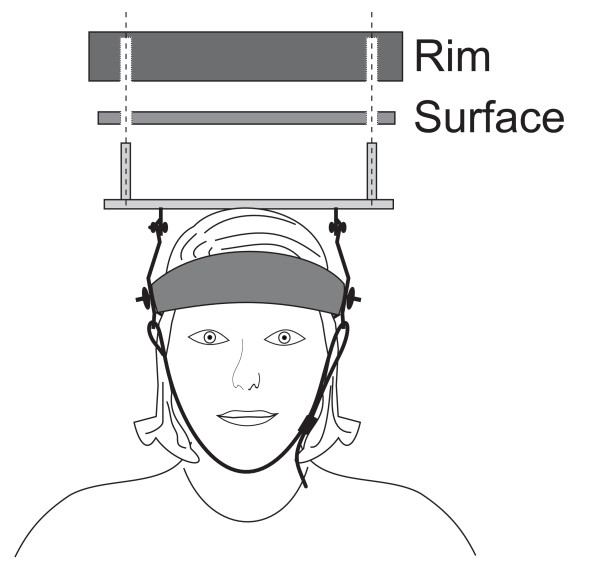
**Exploded drawing of the neck coordination exercise device, including the removable rim and an exchangeable surface**. Four different surfaces were used in order to vary the rolling resistance of the ball.

**Figure 3:**
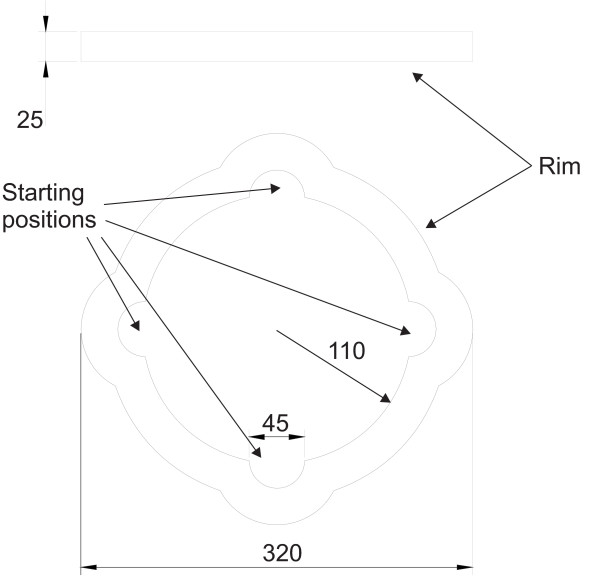
**The removable rim viewed from the side and from above**. Before commencing a trial, the subject should place the ball in a starting position (at front, back, left or right) by tilting the plate. All measurements are in millimetres.

A newly developed device (patent SE serial # 530879) was used for the neck-coordination exercise. The device was designed to allow for an exercise involving an open skills task, task variation, continuous feedback and progression of difficulty (fig. 1). The device consisted of a plate with exchangeable surface (fig. 2) and a removable rim (fig. 3) (weighing altogether 760 gram). Four different surfaces was used to increase difficulty in the following order: fleece fabric (Malden Mills Polartec^® ^Classic 100), cotton fabric, copy paper (80 g/m^2^), all glued on a Plexiglas board, and finally an uncovered Plexiglas board.

The exercise was performed in a functional sitting position in an adjustable office chair. The subject was provided with visual feedback of the device via mirrors. To ensure a good sitting posture during the exercise, the subject was instructed to sit upright with lumbar support and the head balanced in line with the torso, the hips and knees about 90° flexion and the lower arms resting on armrests. In this position the task was to move the metal ball (weight 220 gram) from a starting position, by controlled movements of the head, to the centre of the plate and hold it still for three s (see additional file 1: Movie 1 for the neck coordination exercise.mpg). The three second period was chosen to require that the subjects could fine control the positioning of the ball. The location of the ball at starting and target positions was monitored by LED-photocell detectors. The signals from these detectors were monitored by a PC and allowed for automation of the training, including delivery of pre-recorded verbal instructions. The trunk was fixed to the chair via a strap placed around the subjects torso as seen in Figure 1., in order to promote movements with the neck and head rather than with the trunk. After succeeding with the task, or after 47 s if the task was not successfully completed, the subject was instructed to move the ball to another starting position. Light emitting diodes indicated the starting position and when the ball was at the center of the plate. When the subject had acquired the skill to complete at least five trials in a block of six trials, with each trial completed within 30 s, the difficulty of the task was increased by changing the surface to a faster one (i.e., lower rolling resistance).

The intervention consisted of eight training sessions performed two-three times per week over a time period of four weeks. Each session lasted 10–15 minutes and consisted of three blocks of six trials. The exercise dose and the progression of difficulty were based on a previous pilot study on eight healthy adults (six males), with a mean age of 26. The healthy group performed eight exercise sessions over a four week period. Improvement in exercise performance was measured and the subjects where interviewed after the exercise period. No other measurements where collected. All the healthy subjects showed a rapid improvement in the exercise performance. The interviews revealed an overall positive opinion about the exercise method and no adverse effects were reported other than transient discomfort and tiredness. The most common strategies to succeed with the task were to perform small and slow movements. The supervision of the exercise and the collecting of measurements in the previous and the present pilot studies were executed in a clinical setting by the first author.

### Outcome measures

Measurements collected at baseline were sensorimotor function tests, questionnaires and pain measurements. Post intervention measurements were sensorimotor function tests, pain measurements and subjective experience of the exercise. Six months follow up was accomplished with questionnaires and pain ratings. Throughout the exercise period skill acquisition was measured.

#### Clinical applicability

The acquisition of the skill to perform the neck coordination task was assessed by recording the trial times as well as monitoring the rate of progression to successively faster surfaces during the intervention period. The outcome variable for the improvement in performing the task, was the slope of the linear regression of the trial times on the fastest surface for each subject. Such calculations were not performed for the slower surfaces due to rapid progression, resulting in insufficient data (i.e., to few blocks of trials) for a meaningful regression analysis.

To assess the subjective experiences of the method, the subjects were interviewed after the last training session by the test leader asking predetermined questions. Questions included in the interview were: (1) What is your overall opinion about the training method? (2) How comfortable was the plate on the head? (3) Were the instructions easy to understand? (4) Did you experience tiredness, discomfort or pain from the training? (5) Did you use any strategy when trying to succeed with the exercise task? For each question a few categories were formed on the basis of the replies.

#### Sensorimotor function tests

Tests of postural sway and cervical axial rotation were performed in quiet stance bare footed on a force platform with the feet together, heel-to-heel and toe-to-toe. Instructions were pre-recorded and provided through speakers. The test order was the same for all subjects, starting with postural sway followed by cervical rotation. The selection of tests and the sensorimotor variables calculated from these tests were based on two premises: (1) that they have discriminative value for people with chronic neck pain [2,3,5-8] and (2) that they represent different aspects of sensorimotor control as indicated by weak or absent inter-variable correlations [20,21].

In the postural sway test the subject was instructed to stand as still as possible during 30 s with eyes closed and arms crossed over the chest. Prior to the test a 10-s training session was given. A static force platform (Kistler Force Measurements, type 9807, Kistler Instrumente AG, Switzerland) was used for measuring the centre of pressure (CoP) migration in the anterior-posterior and medio-lateral directions with a sampling frequency of 30 Hz. It is well established that the CoP signal is composed of two main components, one slow and one fast, reflecting separate mechanisms of the postural control system (e.g., [22,23]). We chose to decompose the CoP signals into the slow rambling (Ra) and fast trembling components (Tr) according to the method described by Zatsiorsky and Duarte [22]. As outcome variables we calculated the 95% confidence area of the ellipse of the Ra and Tr trajectories from 24 s of the data, excluding 5 s at the beginning and 1 s at the end of the trial.

The cervical rotation test was performed in two separate conditions in randomized order; rotation to the right and to the left. In each condition, a block of eight consecutive rotations (trials) was executed with a short resting interval between trials. The subject was instructed to rotate the head with a fast movement as far as possible. Prior to each trial the subject was instructed to memorize the starting position, which was a self-selected neutral position of the head, and to reproduce this position as accurately as possible after each head rotation. The instruction given was "memorize your head position", "make a fast rotation of your head as far as possible" and "return to the starting position as accurately as possible". Prior to each block the subject was given a training session of two trials. Cervical kinematics was measured with an electromagnetic tracking system (FASTRAK™, Polhemus Inc, USA), with a sampling rate of 60 Hz. Two receivers, one positioned on the forehead and the other above the dorsal spinal process of Th2, were used for recording the axial head rotation relative to the trunk. A quintic spline (generalized cross-validation spline) [24] was used for low-pass filtering and differentiation of the angular data.

In total, five outcome variables were calculated from the cervical rotation test: The range of movement (*ROM*) was calculated as the maximal angular excursion from the starting (normal) head position. To assess the movement smoothness and speed, the *movement time*, *peak velocity *and *jerk index *were calculated for the rotation from the starting position to maximal excursion angle. The start and stop of the rotation used for these calculations were defined by a threshold of 5% of the peak angular velocity. To estimate the acuity of cervical repositioning we calculated the variable error (*VE*) of repositioning. First, the difference between the reproduced angle (the angle when the angular velocity had remained below 5% of peak velocity for 0.5 s) and the starting angle (0.5 s before the start of the movement) were calculated for each trial (algebraic errors). In order to remove any systematic drift in the errors, which is unrelated to the response variability but will affect VE [25], the algebraic errors were detrended by computing the residuals from a straight-line fit of the errors. Thereafter VE was calculated as the population standard deviation of the error residuals. The *jerk index *was calculated from the third derivative of the head- trunk angle using the algorithms described in Kitazawa et al. [26], which normalizes the jerk cost with respect to movement distance and time. For all calculations, the trials for left and right rotations were pooled after removal of outlier trials (± 2 SD), and mean values were calculated, except for ROM where the maximal value was used.

Test-retest data from other studies which included the same methods as used in the present study was analysed to get an indication of the reliability of the tests (intra class correlation coefficient (ICC)) and to reveal possible retest effects on mean values (paired t-test). The p-values of the t-test and ICC_3,1 _were for postural sway variables *Ra area *p = 0.34 and ICC = 0.51 and for *Tr area *p = 0.81 and ICC = 0.47 (n = 70 mixed group including healthy adults and subjects with chronic neck pain, one week test interval), and for the cervical rotation variables: *jerk index *p = 0.98 and ICC = 0.93, *movement time *p = 0.37 and ICC = 0.93, *peak velocity *p = 0.99 and ICC = 0.91, *ROM *p = 0.039 and ICC = 0.92, *repositioning VE *p = 0.85 and ICC = 0.73 respectively (n = 12 healthy adults, four weeks test interval).

#### Pain and self-reported characteristics

Self-rated pain was assessed as pain at the moment and measured within a week before the day of testing on a blank 100 mm *visual analogue scale *(VAS), on which 0 mm corresponds to "no pain at all" and 100 mm to "worst imaginable pain" [27].

Self-reported characteristics were assessed by the following questionnaires: *The Short Form 36 *(SF-36) was used as a measure of subjective functional health and well being [28]. Severity of symptoms and disability was measured using *the Neck Disability Index *(NDI) [19]. *The TAMPA Scale of Kinesiophobia *(TSK) was used to measure fear of movement and re-injury due to movement [29]. *The Self-Efficacy Scale *(SES) was used to measure confidence in performing activities of daily living[30]. *The Disability of the Arm, Shoulder and Hand *(DASH) questionnaire was used to measure upper extremity disability and symptoms [31]. The indices of the questionnaires were normalized, i.e., expressed as percentage of the maximum score. Note that higher scores denotes more disability in NDI and DASH and greater fear of movement in TSK, whereas higher scores denotes better functional health and well being in SF-36 and higher confidence in own capability in SES.

### Statistics

One sample t-test was used to analyse the slopes of the linear regression of the skill acquisition of performing the exercise task. The presumed independence between the sensorimotor variables was tested by calculating Pearson's correlation coefficient between all variables. For independent (non-correlated) variables, paired t-test were used to assess changes between the pre- and post-intervention measurements. For correlated variables repeated measures MANOVA were used, if this MANOVA was significant, paired t-tests were performed to identify the variables that contributed to the effect. Jerk index-, movement time-, and VE of cervical rotation were log transformed before analysis due to skewed distribution. The VAS measurements were analyzed with the Friedman test and the questionnaires scores with Wilcoxon signed ranks test. All analyses were performed using SPSS 13.0 for Windows (SPSS Inc., Chicago, Illinois, USA). In all statistical tests, p < 0.05 was considered significant.

## Results

### Clinical applicability

**Figure 4:**
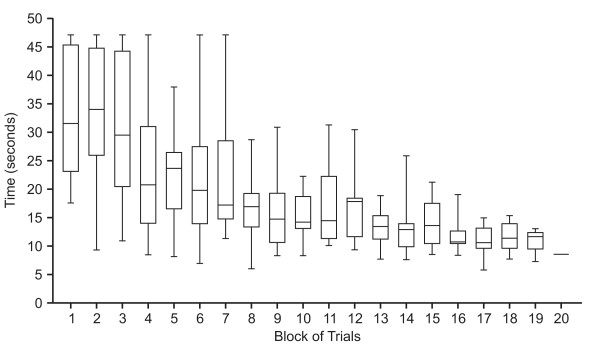
**Performance of the exercise task for the fastest surface during the exercise period**. The figure illustrates the distribution of median trial times over all subjects separately for each block of trials. Due to variation in progression between subjects the number of blocks of trials performed in this condition ranged from 11 to 20. Therefore, for block 1–11 n = 14 and for block 12–20 n = 13, 11, 11, 10, 9, 8, 6, 3 and 1 respectively. For each box plot, the whiskers represent maximum and minimum values, the top and bottom of the box represent the 75th and 25th quartiles and the horizontal line in the box represent the median.

All subjects improved their skill to perform the exercise task. This was evident from the fact that all subjects progressed to the most difficult condition during the intervention period and by the fact that all subjects successively decreased their median time per block (mean slope = -1,4 s/block, one-sample t-test; p < 0.001) on the fastest surface (fig. 4).

Thirteen subjects were interviewed for their experience of the exercise method. One subject was not interviewed due to an oversight by the test leader. The interviews revealed that none of the subjects were negative to the neck coordination exercise. Eleven subjects were positive, while two were neutral. One subject reported discomfort from wearing the device on the head. The comprehension of the instructions was conceived as easy by all the subjects. Seven subjects experienced tiredness, two reported discomfort and four reported pain from the training. Three subjects did not state any symptoms at all connected to the training. A general feature for those who experienced tiredness, discomfort or pain was that the symptoms were transient and mainly present at the first few trainings sessions. However, one subject did experience temporary pain during training throughout the training period. This was the same person who reported discomfort from wearing the device on the head (se above). Five subjects reported post-exercise soreness after the first two-three training sessions. Finally, on the open question about strategies used for the exercise task eight subjects mentioned *deep concentration on the task*, six subjects *to take it easy *and *to perform very small movements of the head*, and four subjects *to relax*.

### Sensorimotor function tests

The correlation analyses of the sensorimotor variables revealed that *repositioning VE *and *ROM *from the cervical rotation test and *Ra and Tr area *from the postural sway test were each uncorrelated to any of the other variables, therefore t-tests were used to assess pre-post intervention changes. Significant decrease was seen for *Tr area *(p = 0.019) (Table 1). The remaining three variables, *jerk index, movement time *and *peak velocity *from the cervical rotation test, were significantly correlated (p < 0.01). These variables were therefore analysed with repeated measure MANOVA, which revealed a significant change (p = 0,045). Since the MANOVA was significant, the dependent variables were analysed with paired t-tests. This analysis indicated that mainly decreased *jerk index *contributed to the effect (p = 0.032, p = 0.320, p = 0.573 for *jerk index*, *movement time *and *peak velocity *respectively).

**Table 1:** Pre- and post intervention data from sensorimotor function tests (n = 14)

	Before intervention Mean (SD)	After intervention Mean (SD)	p-value
Postural sway			
Ra area (cm^2^)	4.27 ± 1.44	3.59 ± 1.79	0.387
Tr area (cm^2^)	1.61 ± 0.67	1.14 ± 0.75	0.019*
Cervical Rotation			
ROM for left + right rotation (degrees)	142 ± 18.3	140 ± 16.7	0.480
VE for cervical repositioning (degrees)	2.54 ± 0.73	3.03 ± 1.41	0.208

Ra: rambling, Tr: trembling, ROM: range of movement, VE: variable error

*p < 0.05.

### Pain and self-reported characteristics

There was no significant decrease in VAS scores after the four-week training period, or at six-months follow up (median and IQR before: 49, 21–75; after: 18, 8–30; follow up: 28, 21–58, p = 0.183. n = 11).

For the SF-36 significant improvement was seen in three out of eight dimensions (Table 2). These were physical functioning, vitality and social functioning. Functional improvement was revealed as decreased disability measured with DASH. Also, fear of movement, measured with TSK, decreased significantly (Table 2).

**Table 2:** Questionnaire scores before intervention and at six-months follow up (n = 12)

	Before intervention Median (IQR)	Follow up Median (IQR)	p-value
NDI	22.0 (16.0–31.5)	15.8, (12.0–33.0)	0.061
SES	94.0, (85,1–96.5)	96.3, (87.8–99.4)	0.415
DASH	31.4, (17.2–40.7)	19.6, (10.3–28.4)	0.038*
TSK	13.8, (11,5–20.2)	7.1, (3.7–15.0)	0.013*
SF-36			
pf	90.0, (75.0–91.3)	90.0, (81.3–95.0)	0.026*
rp	75.0, (37.5–100)	100, (31.3–100)	0.143
bp	51.0, (38,8–64.5)	62.0, (36.8–69.5)	0.310
gh	59.5, (44.3–73.3)	77.0, (56.3–90.8)	0.091
vt	45.0, (30.0–61.25)	67.5, (46.3–80.0)	0.006**
sf	68.8, (46.9–87.5)	100, (75.0–100)	0.007**
re	100, (25.0–100)	100, (75.0–100)	0.496
mh	76.0, (62.0–89.0)	82.0, (80.0–91.0)	0.066
			
pcs	47.6, (38.0–50.4)	48.8, (37.5–52.3)	0.209
mcs	44.6, (32.8–51.0)	52.8, (48.2–55.8)	0.050

Neck Disability Index (NDI), the Self-Efficacy Scale (SES), the Disability of the Arm, Shoulder and Hand (DASH), the TAMPA Scale of Kinesiophobia (TSK) and the Short Form 36 (SF-36) with its eight dimensions: physical functioning (pf), role limitations due to physical problems (rp), bodily pain (bp), general health perceptions (gh), vitality (vt), social functioning (sf), role limitations due to emotional problems (re) and mental health (mh). Physical component summary (pcs) and mental component summary (mcs) represents the summary measures of SF-36.

*p < 0.05, ** p < 0.01.

## Discussion

The results expose positive findings regarding the clinical applicability of the novel neck coordination exercise. Also, indication on possible positive effects were seen in some of the sensorimotor functions and self-rated characteristics.

The exercise regime appeared easy to learn, as all subjects improved their skill to perform the exercise task. This was evident by the progression to the most difficult condition and a successively reduced trial time on each condition during the intervention period. These results confirm that the design of the task and the progression of difficulty were well adapted. Most of the subjects expressed a positive opinion about the exercise, and all subjects reported that the task was easy to understand. The adverse effects due to the exercise that were reported were transient tiredness, discomfort, pain and post-exercise soreness. These side-effects were common and occurred predominantly in the early phase of the intervention period, which can be considered normal reactions due to unaccustomed exercise. Only one person experienced discomfort from wearing the device on the head. Together, these facts support the applicability of the method in a clinical practice, as a conjunction to other interventions, e.g., posture correction, manual therapy, strength and endurance exercises, home exercises etc. Also, a majority of the subjects (8 of 13) mentioned *deep concentration on the task *when interviewed about exercise strategies. This indicates that the exercise involved substantial cognitive effort, which is argued to be an important factor for retention and transfer of motor skills [17].

Improvements in sensorimotor functions were indicated by the significant decrease in *Tr area *(the fast component) of postural sway and *jerk index *(see also below) of cervical rotation. This suggests that there may be a transfer effect from the exercise task to other, non-task specific, motor functions, such as increased postural stability and smoothness of cervical movements. Alternatively, these findings may be simple retest effects. However, as presented in the Methods section, former test-retest data on the same variables revealed no retest effects, which speaks against such an interpretation. In accordance with the present finding, improvements in postural sway has been reported in studies on people with neck pain who received physical therapy which included exercises for cervical muscles [6,32]. A possible explanation to the effects may be improved function of the deep cervical muscles, which are known to contain a high density of muscle spindles, and thereby are important for the postural control. The importance of the proprioceptive input from the neck on the control of posture has been revealed for example in experiments using neck muscle vibration [33]. Moreover, immobilisation of the cervical spine has been associated with unbeneficial effects on sensorimotor functions. Negative effects on eye movements and postural control was reported after a one-week constant use of a cervical collar in a group of healthy people [34]. Since the activity of the deep cervical muscles was not assessed in the present study the possibility of improved proprioceptive input from these muscles remains speculative. The significant repeated measure MANOVA for *jerk index, movement time *and *peak velocity *from the cervical rotation test is somewhat ambiguous to interpret since it is the result of three correlated variables. However, the subsequent paired t-tests indicated that *jerk index *was the main contributor to the effect. Reduced jerkiness after the exercise indicates that the subjects executed the cervical rotation with smoother movements. Less jerkiness is argued to reflect better movement control [35]. The clinical relevance of the effects on postural sway and smoothness of cervical rotations is supported by studies showing increased postural sway during quiet stance in neck pain patients [5,7,8] and recent studies showing increased *jerk index *[3] and irregularities (rate of zero-crossings) [36] during cervical movements in people with chronic neck pain. In contrast to other studies on proprioceptive and coordination training [12,14] no effect was seen in cervical repositioning in the present study. The explanation may be that cervical position sense was not influenced by this exercise, while another possible explanation may reside in the difference in methods used for measuring position sense. In the present study position sense was measured with subjects standing, making fast movements as far as possible while in the studies mentioned above the subjects were seated, performing slow movements. Taken together, these results support the use of measurements of postural sway and cervical kinematics as outcome variables in future randomized control trials.

A relevant question is whether the present sample has impaired sensorimotor functions compared to healthy controls. Comparing the present sample with a group of healthy controls (n = 21) from an unpublished cross-sectional study did not reveal significant reductions in postural control or cervical movement smoothness. Nevertheless, as mentioned above, disturbances in these sensorimotor functions has been documented in previous studies on people with neck pain. Future studies on this novel neck coordination exercise method should therefore preferably include specifically selected samples of people with postural control and movement smoothness disturbances.

The improvements seen in some of the questionnaire scores at the six-months follow up could be either an effect of the intervention or an effect of natural recovery over time. The improvements of the TSK score may indicate that individually adjusted exercise have positive effect on fear of re-injury due to movement. This reasoning is supported by the results presented by Bunketorp et al. [37], showing that supervised individually adjusted physical exercise improved the TSK score in subjects suffering from whiplash associated disorders.

No significant improvements were found for pain ratings (VAS). The reason may be that this exercise has no effect on pain, or that the dosage of the exercise was too small. Earlier studies which have reported decreased pain ratings after intervention involved more frequent training regimes [12,14,15], suggesting that future studies on this method should involve a more extensive intervention period.

This study is limited to investigation of the clinical applicability of the novel exercise for subjects with chronic non-traumatic neck pain in a working age population. Other possible applications that may need further exploration is for rehabilitation of acute neck pain, whiplash associated disorders and diseases that involve dysfunction of the postural control, e.g. vestibular and neurological diseases, as well as fall risk in elderly people. The study has some important limitations concerning the implication of the post intervention measurements. Firstly, the lack of a control group and the relatively small sample size imply that no firm conclusions can be drawn regarding the effects on sensorimotor function tests, questionnaires and pain measurements. Secondly, the tests, exercise instructions and interviews were performed by the same experimenter. Blinded test leaders are needed in future studies. Thirdly, eight training sessions of 10–15 minutes is a relatively small dosage, which implies that the exercise may not have reached full effect. Lastly, the sensitivity of the sensorimotor function tests can likely be improved. Longer sampling periods for the postural sway test would probably improve the reliability of the slow component specifically. Testing the postural sway in different conditions could also be valuable, e.g., with open and closed eyes, on firm and soft surface and by using perturbation, such as vibration of calf and neck muscles and galvanic stimulation of the vestibular nerves. The cervical repositioning test may be more sensitive if performed with slow movements in a sitting position.

## Conclusion

All subjects improved their skill to perform the exercise task. The comprehension of the task was conceived as easy, and a majority expressed a positive opinion about the exercise. Although transient pain and discomfort was common, especially in the early phase of the exercise period, no residual negative side-effects were reported. Taken together, this supports the clinical applicability of the method. The indications on improvements in sensorimotor functions may suggest transfer from the exercise task to other, non-task specific motor functions. The results support a future randomized controlled trial on the exercise effects.

## Competing interests

The study was supported by funding from Alfta Research Foundation. The patent for the novel device is owned by three of the authors and mr Larson, the engineer.

## Authors' contributions

UR participated in the design of the study, carried out the data acquisition, and the statistical analyses and drafted the manuscript. MBJ participated in the design and coordination of the study, the statistical analyses and helped to draft the manuscript. MBE participated in the design and coordination of the study. MD conceived of the study, participated in the design, statistical analyses of the study and helped to draft the manuscript. All authors read and approved the final manuscript.

## Supplementary Material

Additional file 1 Neck coordination exercise. Movie of neck coordination exercise performed with the novel device on a medium fast surface. Click here for file
